# Prähospitale Analgesie mit Nalbuphin und Paracetamol im Vergleich zu Piritramid durch Notfallsanitäter*innen – eine multizentrische Observationsstudie

**DOI:** 10.1007/s00101-024-01449-7

**Published:** 2024-08-23

**Authors:** Marvin Deslandes, Martin Deicke, Julia Johanna Grannemann, Jochen Hinkelbein, Annika Hoyer, Matthias Kalmbach, André Kobiella, Bernd Strickmann, Thomas Plappert, Gerrit Jansen

**Affiliations:** 1 Universitätsklinik für Anästhesiologie, Intensivmedizin und Notfallmedizin, Johannes Wesling Klinikum Minden, Ruhr Universität Bochum, Hans-Nolte-Straße 1, 32429 Minden, Deutschland; 2 Ärztliche Leitung Rettungsdienst, Landkreis Osnabrück, Am Schölerberg 1, 49082 Osnabrück, Deutschland; 3 Klinik für Anästhesiologie, Klinikum Osnabrück, Am Finkenhügel 1, 49076 Osnabrück, Deutschland; 4 Ärztliche Leitung Rettungsdienst, Kreis Gütersloh, Herzebrocker Straße 140, 33324 Gütersloh, Deutschland; 5 Biostatistik und Medizinische Biometrie, Medizinische Fakultät OWL, Universität Bielefeld, Universitätsstraße 25, 33615 Bielefeld, Deutschland; 6 Ärztliche Leitung Rettungsdienst Landkreis Fulda, Fachdienst Gefahrenabwehr, Landkreis Fulda, Otfrid-von-Weißenburg-Str. 3, 36043 Fulda, Deutschland; 7 Zentrale Notaufnahme, Klinikum Fulda, Pacelliallee 4, 36043 Fulda, Deutschland; 8 Malteser Rettungsdienst Hessen, Schmidtstraße 67, 60326 Frankfurt/Main, Deutschland; 9 Stv. Ärztliche Leitung Rettungsdienst, Landkreis Fulda, Otfrid-von-Weißenburg-Str. 3, 36043 Fulda, Deutschland; 10 Medizinische Fakultät OWL, Universität Bielefeld, Universitätsstraße 25, 33615 Bielefeld, Deutschland

**Keywords:** Rettungsdienst, Schmerz, Präklinisch, Notarzt, Notfallsanitäter, Ambulance service, Pain, Prehospital, Emergency doctor, Paramedic

## Abstract

**Fragestellung:**

Angesichts der Änderungen des Betäubungsmittelgesetzes untersucht die vorliegende Arbeit die prähospitale Analgesie durch Notfallsanitäter*innen mittels Piritramid vs. Nalbuphin + Paracetamol.

**Material und Methode:**

Alle prähospitalen Analgesien durch Notfallsanitäter*innen der Rettungsdienste der Kreise Fulda (Piritramid) sowie Gütersloh (Nalbuphin + Paracetamol) wurden im Hinblick auf die Schmerzstärke anhand der Numeric Rating Scale (NRS) zu Einsatzbeginn und -ende sowie die aufgetretenen Komplikationen ausgewertet.

**Ergebnisse:**

Insgesamt wurden 2429 Analgesien ausgewertet (Nalbuphin + Paracetamol: 1635 (67,3 %), NRS-initial: 8,0 ± 1,4, NRS-Einsatzende: 3,7 ± 2,0; Piritramid: 794 (32,7 %), NRS-initial: 8,5 ± 1,1, NRS-Einsatzende: 4,5 ± 1,6). Faktoren mit Einfluss auf eine NRS-Veränderung waren: initiale NRS (Regressionskoeffizient (RK): 0,7075, 95 %-Konfidenzintervall (95 %-KI): 0,6503–0,7647, *p* < 0,001) sowie Therapie mit Nalbuphin + Paracetamol (RK: 0,6048, 95 %-KI: 0,4396–0,7700, *p* < 0,001). Die Therapie mit Nalbuphin + Paracetamol (*n* = 796 (48,7 %)) im Vergleich zu Piritramid (*n* = 190 (23,9 %)) erhöhte die Chancen, eine NRS < 4 am Einsatzende aufzuweisen (Odds Ratio (OR): 2,712, 95 %-KI: 2,227–3,303, *p* < 0,001). Komplikationen traten bei Therapie mit Piritramid bei *n* = 44 (5,5 %) und bei Nalbuphin + Paracetamol bei *n* = 35 (2,1 %) auf. Risikofaktoren für Komplikationen waren Analgesie mit Piritramid (OR: 2,699, 95 %-KI: 1,693–4,301, *p* < 0,001), weibliches Geschlecht (OR: 2,372, 95 %-KI: 1,396–4,029, *p* = 0,0014) sowie das Lebensalter (OR: 1,013, 95 %-KI: 1,002–1,025, *p* = 0,0232).

**Diskussion:**

Im Vergleich mit Piritramid weist die prähospitale Analgesie mit Nalbuphin + Paracetamol günstige Effekte im Hinblick auf analgetische Effektivität und Komplikationsraten auf und sollte in zukünftigen Empfehlungen für Notfallsanitäter*innen berücksichtigt werden.

## Abstract (en)

**Objective:**

Following recent changes to the German Narcotics Act, this article examines prehospital analgesia by paramedics using piritramide vs. nalbuphine + paracetamol.

**Material and methods:**

Prehospital analgesia administered by paramedics from the Fulda (piritramide) and Gütersloh (nalbuphine + paracetamol) emergency services was compared regarding pain intensity at the beginning and end of the mission, measured using the numeric rating scale (NRS). Additionally, an analysis of the resulting complications was carried out.

**Results:**

In this study 2429 administrations of analgesia were evaluated (nalbuphine + paracetamol: 1635, 67.3%, initial NRS: 8.0 ± 1.4, end of NRS: 3.7 ± 2.0; piritramide: 794, 32.7%, initial NRS: 8.5 ± 1.1, end of NRS: 4.5 ± 1.6). Factors influencing NRS change were initial NRS (regression coefficient, RC: 0.7075, 95% confidence interval, CI: 0.6503–0.7647, *p* < 0.001), treatment with nalbuphine + paracetamol (RC: 0.6048, 95% CI: 0.4396–0.7700, *p* < 0.001). Treatment with nalbuphine + paracetamol (*n* = 796 (48.7%)) compared to piritramide (*n* = 190 (23.9%)) increased the odds of achieving NRS < 4 (odds ratio, OR: 2.712, 95% CI: 2.227–3.303, *p* < 0.001). Complications occurred in *n* = 44 (5.5%) with piritramide and in *n* = 35 (2.1%) with nalbuphine + paracetamol. Risk factors for complications were analgesia with piritramide (OR: 2.699, 95% CI: 1.693–4.301, *p* < 0.001), female sex (OR: 2.372, 95% CI: 1.396–4.029, *p* = 0.0014), and age (OR: 1.013, 95% CI: 1.002–1.025, *p* = 0.0232).

**Conclusion:**

Compared with piritramide, prehospital analgesia with nalbuphine + paracetamol has favorable effects in terms of analgesic efficacy and complication rates and should therefore be considered in future recommendations for paramedics.

## Hinführung zum Thema

Obwohl eine frühzeitige suffiziente Analgesie als zentraler Qualitätsindikator der prähospitalen Versorgung gilt und eine Voraussetzung ein bestehendes Analgesiekonzept ist, sind viele Patient*innen weiterhin nur unzureichend versorgt [8, 9, 16, 19, 24]. Als ursächlich hierfür wurde bis zu seiner jüngsten Novellierung u. a. das Fehlen potenter Analgetika für die Verwendung durch Notfallsanitäter*innen angesichts der gesetzlichen Restriktionen durch das Betäubungsmittelgesetz (BtMG) angesehen [4, 5, 12, 19, 21, 22].

## Hintergrund

Am 27.07.2023 wurden das BtMG und das Notfallsanitätergesetz (NotSanG) geändert [13]: Nach Vorgabe eines entsprechenden Analgesiekonzeptes gemäß einer Standard Operating Procedure (SOP) durch eine verantwortliche ärztliche Person dürfen Notfallsanitäter*innen nun auch Analgetika im Sinne der Anlage III BtMG verabreichen, sofern dies zur Gefahrenabwendung für Gesundheit oder zur Beseitigung oder Linderung erheblicher Beschwerden erforderlich ist [15]. Während Befürwortende dieser Änderungen die Effektivität hochpotenter Opioidanalgetika zur Therapie akuter Schmerzen und die Reduktion der Bindung von Notarzt*innen anführen, sehen Kritisierende die potenziell vital bedrohlichen Nebenwirkungen von µ‑Agonisten wie Piritramid, Morphin oder Fentanyl als relevanten Nachteil und befürworten trotz der jüngsten Gesetzesänderung den Einsatz von Nicht-BtM-Alternativen [4, 5, 11, 12, 18, 21, 22, 24, 26]. Nalbuphin demgegenüber weist als κ‑Agonist-µ-Antagonist Vorteile, die für den Einsatz durch Notfallsanitäter*innen prädisponieren, auf: Nach 2‑ bis 3‑minütiger Anschlagszeit besitzt es eine gute Effektivität bei mittel- bis starken Schmerzen verschiedenster Entitäten mit nur geringem Risiko vital bedrohlicher Nebenwirkungen [1, 7, 25, 28]. Trotz dieser Vorteile, und obgleich Nalbuphin im Ergänzungsteil Schmerz der BPR/SAA 2023 der 6‑Länder-Arbeitsgruppe der Ärztlichen Leitungen Rettungsdienst von Baden-Württemberg, Brandenburg, Mecklenburg-Vorpommern, Nordrhein-Westfalen, Sachsen und Sachsen-Anhalt bereits Erwähnung findet, ist der Einsatz von Nalbuphin durch Notfallsanitäter*innen bislang nicht weit verbreitet [14]. Obwohl durch die erfolgte Gesetzesänderung zu erwarten ist, dass prä- und innerklinische Notfallmediziner*innen zukünftig mit durch Notfallsanitäter*innen analgetisch vorbehandelten Patient*innen und therapieassoziierten Komplikationen konfrontiert werden, so fehlen bislang Studien, die eine prähospitale Analgesie durch Notfallsanitäter*innen mit Nalbuphin im Vergleich zu dem verbreiteten µ‑Agonisten Piritramid untersuchen. Daher evaluiert die vorliegende Arbeit die Effektivität und Komplikationen einer prähospitalen Analgesie mit Nalbuphin + Paracetamol im Vergleich zu Piritramid anhand von Rettungsdienstdaten der Kreise Gütersloh und Fulda.

## Material und Methoden

Nach positivem Votum der zuständigen Ethikkommission der Ärztekammer Westfalen-Lippe vom 09.02.2022 (Az. 2022-031-f-S) wurden in einem retrospektiven Studiendesign alle Notfalleinsätze der Rettungsdienste des Kreises Gütersloh (01.01.2020–30.06.2022) sowie des Landkreises Fulda (01.01.2018–31.05.2023) in die Untersuchung einbezogen. Da es sich um eine retrospektive Cluster-Analyse der beiden Studienzentren handelte, sind die Untersuchungszeiträume nicht vollkommen identisch.

### Studienzentren

Der Rettungsdienst des Kreises Gütersloh stellt die Versorgung von ca. 364.000 Menschen im Kreis Gütersloh mit 10 Rettungswachen, 28 Rettungswagen und 5 Notarztstandorten sicher. Die Bevölkerung der ca. 223.500 Menschen im Landkreis Fulda wird durch 13 Rettungswachen mit 27 Rettungswagen, 4 Notarztstandorten und einem Rettungshubschrauber versorgt.

### Studienprotokoll

Durch die Ärztlichen Leitungen Rettungsdienst der Studienzentren erfolgten unabhängig voneinander die Erstellung sowie Einweisung der Notfallsanitäter*innen in eine SOP zum Vorgehen bei behandlungsbedürftigen Schmerzen. Betreffende Patient*innen mit behandlungsbedürftigen Schmerzen wurde in beiden Studienzentren strukturiert nach Vorerkrankungen, Typ und Stärke der Schmerzen entsprechend der Numeric Rating Scale (NRS) (0 = keine Schmerzen, 10 = stärkste Schmerzen) gefragt. Nach Durchführung von Basismaßnahmen (bedürfnisgerechte Lagerung, Anlage einer Venenverweilkanüle und Vollelektrolytlösungsinfusion), Basis-Monitoring (Elektrokardiogramm, pulsoxymetrisch gemessene Sauerstoffsättigung (S_p_O_2_), nichtinvasive Blutdruckmessung) und bedarfsweiser Sauerstoffapplikation (Ziel‑S_p_O_2_ ≥ 94 %) wurde die in den jeweiligen Rettungsdienstbereichen vorgesehene analgetische Therapie begonnen.

### Vorgehen im Rettungsdienst des Kreises Gütersloh

Nach Ausschluss von Kontraindikationen gegen Nalbuphin (Vortherapie mit µ‑Agonisten wie beispielsweise Methadon o. a.) bzw. Paracetamol (z. B. Lebererkrankungen, Allergien u. a.) erhielten die Patient*innen 15 mg/kgKG Paracetamol i.v. bis zu einer Höchstdosis von 1000 mg zusammen mit einer initialen i.v.-Nalbuphin-Dosis von 0,2 mg/kgKG (bei Patient*innen ≥ 65 Jahre 0,1 mg/kgKG Nalbuphin i.v.). Führte dies nicht zu einer ausreichenden Analgesie, war es den Notfallsanitäter*innen möglich, nach einer Mindestwartezeit von 2 min eine einmalige Wiederholungsdosis von 0,1 mg/kgKG (≥ 65 Jahre zweite und dritte Dosis von 0,1 mg/kgKG Nalbuphin) bis zu einer Höchstdosis von 20 mg zu verabreichen.

### Vorgehen im Rettungsdienst des Landkreises Fulda

Im Landkreis Fulda erfolgte nach Ausschluss von Kontraindikationen (Glasgow Coma Scale < 12; Herzfrequenz < 50/min; Atemfrequenz < 10/min; Blutdruck systolisch < 100 mm Hg und S_p_O_2_ < 90 %, Vorliegen einer schweren obstruktiven Lungenerkrankung) die Applikation von Piritramid in einer Dosis von 0,06 mg/kgKG. Bei Fortbestehen der Schmerzsymptomatik waren alle 5–6 min repetitive Piritramidgaben von 0,06 mg/kgKG bis zum Erreichen der vorgegebenen Maximaldosierung von 15 mg möglich.

### Ein- und Ausschlusskriterien

Eingeschlossen wurden alle Patient*innen ≥ 18 Jahre mit behandlungsbedürftigen Schmerzen und analgetischer Therapie durch Notfallsanitäter*innen entsprechend der vorgegebenen SOP mit Nalbuphin + Paracetamol bzw. Piritramid. Ausschlusskriterien waren Patient*innen < 18 Jahre, Abwesenheit behandlungsbedürftiger Schmerzen, keine analgetische Therapie, prähospitale notärztliche Versorgung, Verwendung anderer Analgetika als Nalbuphin + Paracetamol bzw. Piritramid, unvollständige Daten.

### Datenerfassung

Die ausgewerteten Daten entstammen den elektronischen Einsatzdokumentationen bei Patient*innen mit therapiebedürftigen bzw. starken Schmerzen entsprechend der in den jeweiligen Studienorten angewandten SOP. Erfasst wurden Einsatzdatum; Alter; Geschlecht; Schmerzursache [traumatische vs. nichttraumatische Schmerzursachen (muskuloskeletal z.B. nichttraumatische Schmerzen Halswirbelsäule, Thorax oder Knochen und/oder Lumboischialgie), viszeral (abdominelle Schmerzen, Nierenkoliken), nichttraumatischer Brustschmerz, Kopfschmerzen oder andere Schmerzursachen], die Schmerzstärke zu Einsatzbeginn und -ende, gemessen anhand der NRS, ihre Veränderung zwischen Einsatzbeginn und -Ende (∆-NRS), Art und Dosis des applizierten Analgetikums. Darüber hinaus wurden Komplikationen der Analgetikatherapie wie Übelkeit/Erbrechen, Vigilanzreduktion (Glasgow Coma Scale ≤ 14 und/oder Veränderung des mentalen Status des*der Patient*in), respiratorische Insuffizienz (Desaturation, A‑/Bradypnoe, Notwendigkeit einer zusätzlichen Sauerstoffapplikation, assistierten und/oder kontrollierten Beatmung), hämodynamische Insuffizienz (systolischer Blutdruck < 100 mm Hg) erfasst. Primärer Endpunkt war das Erreichen einer adäquaten Analgesie, definiert als NRS < 4 am Einsatzende in Abhängigkeit von den applizierten Opioiden. Sekundäre Endpunkte umfassten die Veränderung der NRS zwischen Einsatzende und -beginn sowie das Auftreten von Komplikationen.

### Statistische Analyse

Zur Analyse des primären Endpunkts wurde eine logistische Regression durchgeführt. Einflussgrößen waren das verwendete Analgesiekonzept (Piritramid vs. Nalbuphin + Paracetamol; Exposition), Alter, Geschlecht, initiale NRS und die Schmerzursache. Ein lineares Regressionsmodell mit den Kovariablen Alter, Geschlecht, initiale NRS und der Schmerzursache wurde verwendet, um potenzielle Assoziationen zwischen der Exposition und dem ∆‑NRS zu untersuchen. Zur Analyse des sekundären Endpunkts „Auftreten von Komplikationen“ wurde eine logistische Regression mit den Kovariablen Analgetika-Exposition, Alter, Geschlecht und Schmerzursache durchgeführt. Die Ergebnisse der logistischen Regressionen wurden anhand von Odds Ratios (OR), die der linearen Regression hingegen anhand von Regressionskoeffizienten (RK), jeweils mit zugehörigen 95 %-Konfidenzintervallen (95 %-KI) und *p*-Werten angegeben. Alle Analysen wurden mit der Software SAS 9.4 (SAS Institute Inc., Cary, NC) durchgeführt.

## Ergebnisse

Ausgewertet wurden alle Notfalleinsätze des Rettungsdienstes des Kreises Gütersloh (*n* = 95.668) sowie des Landkreises Fulda (*n* = 112.655). Eine Therapie mit Nalbuphin + Paracetamol erfolgte bei 1635 (NRS-initial: 8,0 ± 1,4, Median: 8,0 (Q1–Q3: 7,0–9,0)), mit Piritramid bei 794 Patient*innen (NRS-initial: 8,5 ± 1,1, Median: 8,0 (Q1–Q3: 8,0–9,0)).

Die Charakteristika der untersuchten Patient*innen zeigt Tab. 1, Tab. 2 stellt die Patient*innencharakeristika entsprechend der NRS bei Krankenhausübergabe dar.

**Tab. 1:** Patient*innencharakteristika

Variable	Gesamt	Nalbuphin + Paracetamol	Piritramid
	(*n* = 2429)	(*n* = 1635)	(*n* = 794)
	(*n* (%))	(*n* (%))	(*n* (%))
**Alter (Jahre) [±** **SD]**	55,9 ± 21,5	55,1 ± 21,4	57,5 ± 21,7
**∑ Dosis Nalbuphin in mg [±** **SD]**	8,1 ± 8,4	8,1 ± 8,4	–
**∑ Dosis Paracetamol in mg [±** **SD]**	990,50 ± 85,4	990,5 ± 85,4	–
**∑ Dosis Piritramid in mg [±** **SD]**	7,2 ± 3,9	–	7,2 ± 3,6
**Weibliches Geschlecht**	1295 (53,3)	877 (53,6)	418 (52,6)
**Übelkeit und Erbrechen**	67 (2,8)	35 (2,1)	32 (4,0)
**Vigilanzminderung**	4 (0,2)	0 (0,0)	4 (0,5)
**Respiratorische Insuffizienz**	1 (0,04)	0 (0,0)	1 (0,1)
**Hämodynamische Insuffizienz**	7 (0,3)	0 (0,0)	7 (0,9)
**Schmerzursache**			
*Traumatisch*	1221 (50,3)	710 (43,4)	511 (64,4)
*Nichttraumatisch*			
Muskuloskeletal	477 (19,6)	327 (20,0)	150 (18,9)
Viszeral	648 (26,7)	553 (33,8)	95 (12,0)
Nichttraumatischer Brustschmerz	6 (0,2)	0 (0,0)	6 (0,8)
Kopfschmerzen	39 (1,6)	38 (2,3)	1 (0,1)
Sonstige	38 (1,6)	7 (0,4)	31 (3,9)

*SD* Standardabweichung

**Tab. 2:** Patient*innencharakteristika gruppiert entsprechend der Numeric Rating Scale am Einsatzende

	NRS am Einsatzende < 4			NRS am Einsatzende 4–5			NRS am Einsatzende ≥ 6		
Variable	Gesamt	Nalbuphin + Paracetamol	Piritramid	Gesamt	Nalbuphin + Paracetamol	Piritramid	Gesamt	Nalbuphin + Paracetamol	Piritramid
	(*n* = 986)	(*n* = 796)	(*n* = 190)	(*n* = 1017)	(*n* = 576)	(*n* = 441)	(*n* = 426)	(*n* = 263)	(*n* = 163)
	(*n* (%))	(*n* (%))	(*n* (%))	(*n* (%))	(*n* (%))	(*n* (%))	(*n* (%))	(*n* (%))	(*n* (%))
**Alter in Jahren (MW** **±** **SD)**	55,7 ± 21,3	55,2 ± 21,0	57,5 ± 22,7	56,0 ± 22,2	55,1 ± 22,7	57,2 ± 22,5	56,3 ± 20,1	55,1 ± 20,7	58,2 ± 18,9
**Initiale NRS**									
*MW* *±* *SD*	7,8 ± 1,4	7,8 ± 1,5	8,0 ± 1,2	8,2 ± 1,2	7,9 ± 1,3	8,4 ± 1,0	8,7 ± 1,1	8,5 ± 1,2	9,1 ± 1,0
*Median (Q1; Q3)*	8,0 (7,0; 9,0)	8,0 (7,0; 9,0)	8,0 (7,0; 9,0)	8,0 (7,0; 9,0)	8,0 (8,0; 10,0)	8,0 (8,0; 9,0)	9,0 (8,0; 10,0)	8,0 (8,0; 10,0)	9,0 (8,0; 10,0)
**NRS bei Übergabe**									
*MW* *±* *SD*	2,2 ± 1,0	2,1 ± 1,0	2,6 ± 0,6	4,4 ± 0,5	4,4 ± 0,5	4,4 ± 0,5	7,0 ± 1,2	7,1 ± 1,2	6,9 ± 1,2
*Median (Q1; Q3)*	2,0 (2,0; 3,0)	2,0 (2,0; 3,0)	3,0 (2,0; 3,0)	4,0 (4,0; 5,0)	4,0 (4,0; 5,0)	4,0 (4,0; 5,0)	7,0 (6,0; 8,0)	7,0 (6,0; 8,0)	6,0 (6,0; 7,0)
**NRS-Veränderung**									
*MW* *±* *SD*	5,6 ± 1,8	5,7 ± 1,9	5,4 ± 1,3	3,7 ± 1,3	3,5 ± 1,4	4,0 ± 1,1	1,7 ± 1,4	1,4 ± 1,4	2,2 ± 1,2
*Median (Q1; Q3)*	6,0 (4,0; 7,0)	6,0 (4,0; 7,0)	5,0 (5,0; 6,0)	4,0 (3,0; 5,0)	4,0 (3,0; 4,0)	4,0 (3,0; 5,0)	2,0 (0,0; 3,0)	1,0 (0,0; 2,0)	2,0 (1,0; 3,0)
**Applizierte Dosis (∑)**									
*Nalbuphin (mg)*	8,7 ± 8,2	8,7 ± 8,2	–	7,7 ± 8,3	7,7 ± 8,3	–	6,9 ± 9,0	6,9 ± 9,0	–
*Paracetamol (mg)*	991,2 ± 83,3	991,2 ± 83,3	–	988,5 ± 89,4	988,5 ± 89,4	–	992,8 ± 82,8	992,8 ± 82,8	–
*Piritramid (mg)*	6,8 ± 3,5	–	6,8 ± 3,5	6,9 ± 3,3	–	6,8 ± 3,5	8,6 ± 4,0	–	8,6 ± 4,0
**Weibliches Geschlecht**	511 (51,8)	414 (52,0)	97 (51,0)	565 (55,6)	320 (55,6)	245 (55,6)	219 (51,4)	143 (54,4)	76 (46,6)
**Nausea et vomitus**	22 (2,2)	17 (2,1)	5 (2,6)	32 (3,1)	11 (1,9)	21 (4,8)	13 (3,0)	7 (2,7)	6 (3,7)
**Vigilanzminderung**	1 (0,1)	0 (0,0)	1 (0,5)	3 (0,3)	0 (0,0)	3 (0,7)	0 (0,0)	0 (0,0)	0 (0,0)
**Respiratorische Insuffizienz**	0 (0,0)	0 (0,0)	0 (0,0)	1 (0,1)	0 (0,0)	1 (0,2)	0 (0,0)	0 (0,0)	0 (0,0)
**Hämodynamische Insuffizienz**	3 (0,3)	0 (0,0)	3 (1,6)	2 (0,2)	0 (0,0)	2 (0,4)	2 (0,5)	0 (0,0)	2 (1,2)
**Schmerzursache**									
*Traumatisch*	475 (48,2)	337 (42,3)	138 (72,6)	551 (54,2)	264 (45,8)	287 (65,1)	195 (45,8)	109 (41,4)	86 (52,8)
*Nichttraumatisch*									
Muskuloskeletal	179 (18,1)	151 (19,0)	28 (14,7)	209 (20,5)	123 (21,3)	86 (19,5)	89 (20,9)	53 (20,1)	36 (22,1)
Viszeral	304 (30,8)	290 (36,4)	14 (7,4)	228 (22,4)	175 (30,4)	53 (12,0)	116 (27,2)	88 (33,5)	28 (17,2)
Nichttraumatischer Brustschmerz	1 (0,1)	0 (0,0)	1 (0,5)	3 (0,3)	0 (0,0)	3 (0,7)	2 (0,5)	0 (0,0)	2 (1,2)
Kopfschmerzen	16 (1,6)	15 (1,9)	1 (0,5)	13 (1,3)	13 (2,3)	0 (0,0)	10 (2,3)	10 (3,8)	0 (0,0)
Sonstige	11 (1,1)	3 (0,4)	8 (4,2)	13 (1,3)	1 (0,2)	12 (2,7)	14 (3,3)	3 (1,1)	11 (6,7)

*MW* Mittelwert, *NRS* nummerische Rating-Skala, *SD* Standardabweichung

Bei Übergabe im Krankenhaus betrug der NRS-Mittelwert nach Therapie mit Nalbuphin + Paracetamol 3,7 ± 2,0 (Median: 4,0; Q1–Q3: 2,0–5,0; ∆‑NRS: Mittelwert:4,2 ± 2,3; Median: 4,0, Q1–Q3: 3,0–6,0), nach Anwendung von Piritramid 4,5 ± 1,6 (Median: 4,0, Q1–Q3: 4,0–5,0; ∆‑NRS: Mittelwert: 4,02 ± 1,6; Median: 4,0, Q1–Q3: 3,0–5,0).

Eine NRS < 4 am Einsatzende wiesen 40,6 % (*n* = 986; Nalbuphin + Paracetamol: *n* = 796 (48,7 %), Piritramid: *n* = 190 (23,9 %)), NRS = 4–5: 41,9 % (*n* = 1107; Nalbuphin + Paracetamol: *n* = 576 (35,2 %), Piritramid: *n* = 441 (55,5 %)) und NRS ≥ 6 (17,5 %) (*n* = 426; Nalbuphin + Paracetamol: *n* = 236 (14,4 %), Piritramid: *n* = 163 (20,5 %)) auf.

Die Ergebnisse der logistischen Regressionsanalyse für das Erreichen einer NRS < 4 am Einsatzende sind in der Tab. 3 dargestellt. Patient*innen, die eine Analgesie mittels Nalbuphin + Paracetamol erhielten, hatten höhere Chancen für das Erreichen einer NRS < 4 als Patient*innen, die Piritramid erhielten (OR: 2,712; 95 %-KI: 2,227–3,303; *p* < 0,001).

**Tab. 3:** Ergebnisse der logistischen Regressionsanalyse für das Behandlungsergebnis, nummerische Rating-Skala bei Einsatzende < 4 vs. ≥ 4

Kovariable	Odds Ratio	95 %-Konfidenzintervall	*p*-Wert
Alter	1,001	0,997–1,005	0,7562
Geschlecht (weiblich vs. männlich)	0,908	0,764–1,080	0,2767
*Initiale Numeric Rating Scale*	*0,798*	*0,747–0,851*	*<* *0,001*
*Nalbuphin* *+* *Paracetamol vs. Piritramid*	*2,712*	*2,227–3,303*	*<* *0,001*
Schmerzursache (traumatisch vs. nichttraumatisch)	0,947	0,793–1,132	0,5512

Faktoren mit Einfluss auf eine NRS-Veränderung waren Höhe der NRS-initial (RK: 0,7075; 95 %-KI:0,6503–0,7647; *p* < 0,001) sowie Therapie mit Nalbuphin + Paracetamol (RK: 0,6048, 95 %-KI:0,4396–0,7700, *p* < 0,001) (Tab. 4).

**Tab. 4:** Ergebnisse der logistischen Regressionsanalyse für das Behandlungsergebnis, Veränderung der nummerischen Rating Skala

Kovariable	Regressionskoeffizient	95 %-Konfidenzintervall	*p*-Wert
Alter	−0,00123	−0,00485–0,002385	0,5043
Geschlecht (weiblich vs. männlich)	0,02338	−0,1298–0,1766	0,7648
*Initiale Numeric Rating Scale*	*0,7075*	*0,6503–0,7647*	*<* *0,001*
*Nalbuphin* *+* *Paracetamol vs. Piritramid*	*0,6048*	*0,4396–0,7700*	*<* *0,001*
Schmerzursache (traumatisch vs. nichttraumatisch)	−0,07059	−0,2277–0,08650	0,3783

Analgetikaassoziierte Komplikationen wurden bei 79 Patient*innen (3,2 %; Nalbuphin + Paracetamol: *n* = 35 (2,1 %) vs. Piritramid: *n* = 44 (5,5 %)) beobachtet (Tab. 1). Die logistische Regressionsanalyse zeigte für das Auftreten von Komplikationen ein geringeres Risiko bei Therapie mit Nalbuphin + Paracetamol (OR: 0,371; 95 %-KI:0,323–0,591; *p* < 0,001), weibliches Geschlecht (OR: 2,372; 95 %-KI: 1,396–4,029; *p* = 0,0014) sowie eine Abhängigkeit vom Alter (OR: 1,013; 95 %-KI: 1,002–1,025; *p* = 0,0232) (Tab. 5).

**Tab. 5:** Ergebnisse der logistischen Regressionsanalyse für das Behandlungsergebnis, Auftreten von Komplikationen ja vs. nein

Kovariable	Odds Ratio	95 %-Konfidenzintervall	*p*-Wert
*Alter*	*1,013*	*1,002–1,025*	*0,0232*
*Geschlecht (weiblich vs. männlich)*	*2,372*	*1,396–4,029*	*0,0014*
*Piritramid vs. Nalbuphin* *+* *Paracetamol*	*2,699*	*1,693–4,301*	*<* *0,0001*
Schmerzursache (traumatisch vs. nichttraumatisch)	0,871	0,534–1,420	0,5789

## Diskussion

Die vorliegende Arbeit evaluiert die Effektivität und Komplikationen einer analgetischen Therapie durch Notfallsanitäter*innen mit Nalbuphin + Paracetamol im Vergleich zu Piritramid. Bei gleichen initialen Schmerzniveaus wiesen Patient*innen nach Therapie durch Nalbuphin + Paracetamol im Vergleich zu Piritramid niedrigere NRS und eine höhere Chance auf, eine NRS < 4 bereits prähospital zu erreichen. Faktoren mit günstigem Einfluss auf eine NRS-Veränderung waren die Höhe der initialen NRS und eine Therapie mit Nalbuphin + Paracetamol. Komplikationen waren insgesamt selten und betrafen überwiegend Übelkeit und Erbrechen. Risikofaktoren für Komplikationen waren eine Analgesie mit Piritramid, weibliches Geschlecht sowie das Patient*innenalter.

### Piritramid vs. Nalbuphin + Paracetamol

Während Piritramid und auch Morphin im deutschen Sprachraum sowohl in der Klinik als auch in der Notfallrettung regelmäßige Anwendungen finden, wird Nalbuphin in den letzten Jahren zunehmend durch Notfallsanitäter*innen eingesetzt und hat zu kontrovers geführten Diskussionen geführt [4, 22, 27]: Während Kritisierende die geringere Wirkstärke im Vergleich zu Opioiden wie z. B. Fentanyl und ggf. Entzugssymptome bei Patient*innen mit vorbestehender Opioidtherapie anführen, so sehen Befürwortende des Einsatzes von Nalbuphin besondere Vorteile in der Pharmakokinetik sowie einer bis dato vermuteten geringen Rate an vital bedrohlichen Komplikationen [3, 20]. Die vorliegende Arbeit zeigte durch Nalbuphin + Paracetamol eine höhere Chance, bei Krankenhausaufnahme eine NRS < 4 zu erreichen, obgleich Nalbuphin von manchen Autor*innen den schwach bis mittelstarken Opioiden zugeordnet wird. Hierfür kommen verschiedene Ursachen in Betracht: Erstens, obgleich die applizierten, mittleren Analgetikadosen (Piritramid: 7,2 ± 3,9 mg, Nalbuphin: 8,1 ± 8,4 mg) nahezu identisch waren, ist es durch die Varianz der analgetischen Potenzen von Piritramid und Nalbuphin (Morphinäquivalenz: 0,7–0,75 vs. 0,7–1,1) möglich, dass durch eine Steigerung der Piritramiddosierung ein stärkerer Effekt zu erreichen gewesen wäre. Zweitens, die Anschlagszeiten (Piritramid ≤ 16,8 min vs. Nalbuphin ≤ 3 min) sowie die Zeiten zum Erreichen des Wirkmaximums (Piritramid ≤ 45 min vs. Nalbuphin ca. 10 min) bei vergleichbarer Wirkdauer von ca. 3–6 h weisen darauf hin, dass ein suffizienter analgetischer Effekt durch Piritramid möglicherweise nicht vergleichbar rasch zu erreichen ist [1, 3, 7, 25, 27]. Drittens, während der Anteil der Patient*innen mit viszeraler Schmerzursache in der Therapiegruppe mit Nalbuphin + Paracetamol häufiger war – Nalbuphin weist nur geringe propulsive Effekte auf die glatte Muskulatur viszeraler Organe auf, waren traumatische Schmerzursachen, bei denen eine prähospitale Oligoanalgesie häufiger beobachtet wird, in der Piritramidgruppe häufiger [6, 10, 11, 20, 29]. Viertens, synergistische, opioidsparende Effekte der Kombination des Nalbuphins mit Paracetamol könnten die Ergebnisse beeinflusst haben: Während für die Kombination von nichtsteroidalen Antirheumatika („non-steroidal anti-inflammatory drug“, NSAID) und Metamizol mit Opioiden additive analgetische Effekte nachgewiesen werden konnten, ist die Datenlage bei Einsatz von Paracetamol weiterhin uneinheitlich [30]: Während für Notfallpatient*innen in den Notaufnahmen kein opioidsparender Effekt durch Paracetamol nachgewiesen werden konnte [2], zeigten perioperative Studien sowohl reduzierte Morphindosen als auch das Fehlen eines additiven, analgetischen Effekts [10]. Obgleich in der Gruppe der Patient*innen mit einer analgetischen Therapie mit Nalbuphin + Paracetamol ein deutlich höherer Anteil der Patient*innen am Einsatzende eine NRS < 4 aufwies (Nalbuphin 48,7 % vs. Piritramid 23,9 %, Tab. 2), sollten die evaluierten SOP zukünftig weiter optimiert werden, um präklinisch eine möglichst optimale Schmerztherapie zu gewährleisten.

### Komplikationen der analgetischen Therapie

Beim Vergleich der verbreiteten Opioide Morphin und Fentanyl zeigten sich neben vergleichbaren analgetischen Effekten mit ca. 18 % identische Komplikationsraten [11, 17, 23]. Neben vital bedrohlichen Komplikationen wie respiratorischer Insuffizienz (Fentanyl bis 16,1 %, Morphin bis 4,8 %) mit Notwendigkeit zur assistierten Beatmung (bei Fentanyl bei 0,02 %), Hypotensionen (Fentanyl in 1,6 % vs. Morphin in 0,5 %) traten häufig Übelkeit und Erbrechen (Fentanyl in 1,5 % vs. Morphin in 4,8 %) auf [11]. Für die Infusion von 7,5 mg Piritramid durch Notfallsanitäter*innen beobachtete eine deutsche Studie eine effektive Analgesie, jedoch waren auch hier in Einzelfällen Atemwegsinterventionen notwendig [22]. Während sich auch für die in der vorliegenden Studie untersuchten Analgesiekonzepte eine effiziente Schmerzsenkung mit insgesamt niedrigen Komplikationsraten zeigten, traten respiratorische oder hämodynamische Insuffizienzen ausschließlich nach Piritramidapplikation auf. Eine Therapie mit Nalbuphin + Paracetamol könnte daher zukünftig ein geeignetes, komplikationsarmes Analgesiekonzept nicht nur für Notfallsanitäter*innen darstellen.

### Aspekte der Rechtssicherheit

Zwar hat der Bundesgesetzgeber die grundsätzliche Möglichkeit geschaffen, dass Notfallsanitäter*innen Betäubungsmittel eigenständig anwenden. Dennoch müssen bei der gesetzesgemäßen Umsetzung strengere Voraussetzungen erfüllt sein, als dies bei Anwendung von nicht in Anlage III des BtMG gelisteten Medikamenten der Fall ist. Das BtMG enthält darüber hinaus auch nach der Änderung noch immer die Restriktion, dass die Anwendung (von Betäubungsmitteln) insbesondere dann nicht begründet ist, wenn der beabsichtigte Zweck auf andere Weise erreicht werden kann (§ 13 Absatz 1 Satz 2 BtMG). Die vorliegenden Daten werfen daher die Frage auf, ob eine Applikation der in Anlage III des BtMG gelisteten Analgetika wie z. B. Piritramid gesetzeskonform erfolgen kann, wenn vergleichbare Effekte mit Nalbuphin (kein BtM) zu erzielen sind. Zur abschließenden Klärung dieser Frage sollten zukünftig prospektive randomisierte kontrollierte Studien äquipotente Dosierungsregime von Nalbuphin im Vergleich zu Piritramid evaluieren.

### Limitationen

Die Limitationen der vorliegenden Arbeit umfassen im Wesentlichen die Limitationen retrospektiver Observationsstudien mit entsprechend möglicher Verzerrung der Ergebnisse durch mitunter unterschiedliche Charakteristika, Untersuchungszeiträume und Größe der ausgewerteten Gruppen (Tab. 1). Das Bias-Risiko ist jedoch vermindert, da in der vorliegenden Analyse für potenzielle Confounder adjustiert wurde, jedes der getesteten Analgesiekonzepte an jeweils einem der beteiligten Rettungsdienstbereiche eingesetzt wurde und außerdem ein Hawthorne-Effekt umgangen werden konnte. Nichtsdestotrotz sollten zukünftig prospektive randomisierte Studien durchgeführt werden, um strukturgleiche Gruppen zu evaluieren und ebenfalls die Rolle von Nalbuphin im Vergleich zu anderen Analgetika bei traumatischen Schmerzursachen zu evaluieren. Eine differenziertere Betrachtung des zeitlichen Verlaufes von Effektivität und Komplikationen der untersuchten Analgesiekonzepte war nicht möglich, sodass nur kumulative Analgetikadosen erfasst wurden, weswegen eine Aussage zur Notwendigkeit repetitiver Analgetikaapplikationen nicht möglich ist. Trotzdem erlauben die Ergebnisse erstmalig einen wichtigen Einblick zur Entwicklung komplikationsarmer und effektiver Therapieoptionen angesichts des zu erwartenden häufigeren Einsatzes von Betäubungsmitteln durch Notfallsanitäter*innen. Das prähospitale Studiendesign erlaubt keine Aussage über mögliche Wechselwirkungen des Nalbuphins mit µ‑Rezeptor-Agonisten im innerklinischen Behandlungsverlauf. Zwar wird ein Wechsel von µ‑Rezeptor-Agonisten auf Nalbuphin nicht empfohlen, jedoch besteht bei ineffizienter Nalbuphinwirkung durch kompetitiven Antagonismus die Möglichkeit ihres Einsatzes [3]. Die hier teilweise höheren Dosierungen machen jedoch eine sorgfältige Titration unter kontinuierlichem Monitoring erforderlich [3]. Nalbuphin ist nicht geeignet, µ‑Rezeptor-Agonisten im Rahmen der präklinischen Notfallmedizin zu ersetzen, sondern stellt insbesondere für Rettungsdienstbereiche, in denen die Freigabe für µ‑Rezeptor-Agonisten durch die Ärztlichen Leitungen Rettungsdienst nicht erfolgt, eine gut geeignete Alternative und Ergänzung des Medikamentenrepertoires von Rettungswagen zur effektiven Analgesie dar.

## Fazit für die Praxis

- Patient*innen nach prähospitaler Therapie entsprechend den untersuchten Vorgehensweisen mit Nalbuphin + Paracetamol weisen im Vergleich zu Piritramid niedrigere NRS und eine höhere Chance auf, eine NRS < 4 bereits prähospital zu erreichen.
- Komplikationen waren insgesamt selten und betrafen überwiegend Übelkeit und Erbrechen.
- Risikofaktoren für Komplikationen waren eine Analgesie mit Piritramid, weibliches Geschlecht sowie das Patient*innenalter.
- Zukünftige Studien sollten Konzepte zur prähospitalen Analgesie mit Nalbuphin in Kombination mit Paracetamol im Vergleich zu anderen µ‑Rezeptor-Agonisten anhand von prospektiven Studien evaluieren.
- Die prähospitale Analgesie durch Notfallsanitäter*innen mit Nalbuphin in Kombination mit Paracetamol im Vergleich zu Piritramid erlaubt eine sichere und effektive Analgesie.
